# Prevalence and correlates of induced abortion: results of a facility-based cross-sectional survey of parturient women living with HIV in South Africa

**DOI:** 10.1186/s12978-022-01520-9

**Published:** 2022-12-05

**Authors:** Anthony Idowu Ajayi, Bright Opoku Ahinkorah, Abdul-Aziz Seidu, Oladele Vincent Adeniyi

**Affiliations:** 1 Sexual, Reproductive, Maternal, New-born, Child and Adolescent Health (SRMNCAH) Unit, African Population and Health Research Center, APHRC Campus, Manga Close, Nairobi, Kenya; 2 School of Public Health, Faculty of Health, University of Technology, Sydney, Australia; 3 Centre for Gender and Advocacy, Takoradi Technical University, Takoradi, Ghana; 4 College of Public Health, Medical and Veterinary Sciences, James Cook University, Townsville, QLD Australia; 5 Department of Family Medicine, East London Hospital Complex, Walter Sisulu University/Cecilia Makiwane Hospital, East London, South Africa

**Keywords:** Induced abortion, Pregnancy termination, Unwanted pregnancy, Unintended pregnancy, Contraception, Women living with HIV, South Africa

## Abstract

**Background:**

There is a paucity of studies examining the prevalence and correlates of induced abortion among women living with HIV. Our study fills this gap by examining the prevalence and correlates of induced abortion among parturient women living with HIV in Eastern Cape, South Africa.

**Methods:**

We analysed cross-sectional survey data of the East London Prospective Cohort Study, which took place between September 2015 and May 2016 in three large maternity facilities in the Buffalo/Amathole districts of the Eastern Cape Province of South Africa. A total of 1709 parturient women living with HIV who gave birth over the study period were recruited. We carried out descriptive and inferential statistics.

**Results:**

The prevalence of induced abortion was 19% (95% CI: 17.2–20.9%), but varied by women’s socio-demographic characteristics. Induced abortion prevalence was higher among women aged 25 years and over (21.4%), than among women aged less than 25 years (11.0%). Those ever married or cohabiting (26.8%) reported a higher level of induced abortion than those never-married women (15.6%). Those already diagnosed HIV positive before their index pregnancy (20.2%) had a higher prevalence of induced abortion than those diagnosed during their index pregnancy (14.1%). In the adjusted logistic regression, women were more likely to have ever induced abortion if they were ever married or cohabiting (aOR; 1.86 95% CI; 1.43–2.41), ever smoked (aOR: 1.51; 95% CI: 1.01–2.28) and diagnosed with HIV before their index pregnancy (aOR:1.44; 95% CI: 1.02–2.05) but less likely if younger than 25 years (aOR; 0.51 95% CI:0.35–0.73).

**Conclusion:**

About one in five women living with HIV had ever induced abortion in the study settings, indicating that abortion service is one of the main reproductive health services needed by women living with HIV in South Africa. This is an indication that the need for abortion is somewhat high in this group of women. The finding, therefore, highlights the need for concerted efforts from all stakeholders to address the unmet need for contraception among women living with HIV to prevent unintended pregnancy.

## Plain language summary

There is a paucity of studies examining the prevalence and correlates of induced abortion among women living with HIV. Our study fills this gap by examining the prevalence and correlates of induced abortion among parturient women living with HIV in Eastern Cape, South Africa. We analysed a cross-sectional survey data of the East London Prospective Cohort Study, which took place between September 2015 and May 2016 in three large maternity facilities in the Buffalo/Amathole districts of the Eastern Cape Province of South Africa. A total of 1709 parturient women living with HIV who gave birth over the study period were recruited. We carried out descriptive and inferential statistics. The prevalence of induced abortion was 19%, but varied by women’s sociodemographic characteristics. Induced abortion prevalence was higher among women aged 25 years and over (21.4%), ever married (26.8%), those already diagnosed HIV positive before their index pregnancy (20.2%). About one in five women living with HIV had ever induced abortion in the study settings, indicating that abortion service is one of the main reproductive health services needed by women living with HIV in South Africa. This is an indication that the need for abortion is somewhat high in this women. The finding, therefore, highlights the need for concerted efforts from all stakeholders to address the unmet need for contraception among women living with HIV to prevent unintended pregnancy.

## Introduction

According to the 2020 UNAIDS report on the global AIDS epidemic [1], women constitute 53% of all adults living with HIV. These women face several reproductive health challenges, including unmet need for contraception and unintended pregnancies [2, 3]. In sub-Saharan Africa, where HIV is endemic, 63% of women have an unmet need for sexual and reproductive health services [4]. Other studies have also found low desire for children among women living with HIV due to fear of giving birth to HIV-positive children [5–7]. According to UNAIDS estimates, unintended pregnancy rates among women living with HIV range from 51 to 90% globally [8]. Furthermore, studies from sub-Saharan Africa show that the rates of unintended pregnancies among women living with HIV range from 35 to 71% [9–13]. In South Africa, more than two-thirds of pregnancies among women living with HIV are unintended [14–16], with a higher prevalence among parturient women diagnosed with HIV during pregnancy [16]. Studies comparing the prevalence of unintended pregnancy between women living with or without HIV appear to suggest that those with HIV have a higher prevalence of unintended pregnancy [10, 13].

High unmet need for contraception and contraceptive failure are critical risk factors for unintended pregnancy among women living with HIV. Studies in South Africa have found contraceptive failure due, incorrect and inconsistent condom use, and lack of knowledge of emergency contraception as reasons for the high prevalence of unintended pregnancies among women living with HIV in the country [17, 18]. In a study conducted in Zimbabwe, 48% of women with intended pregnancies were using contraceptives at the time of their conception [13]. Another study shows that four in five women reporting contraceptive failure were using condoms, 13% oral contraceptives, and 5% injectable contraceptives [12].

The high prevalence of unintended pregnancies among women living with HIV necessitate improved access to comprehensive abortion services if requested by women. Comprehensive abortion services offer immense benefits to pregnant women, including saving lives that could have been lost due to unsafe abortions and prevention of temporary or permanent disabilities [19]. While non-use of contraceptives or contraceptive failure may result in unwanted or mistimed pregnancies, pregnant women living with HIV may want induced abortion because of fear of transmitting the virus to the baby [20, 21], fear of exposing the child to later stigma and discrimination, fear of leaving a child orphaned [20, 21] and fear of bringing financial burden on her in relation to the care of herself and the child [22]. Since unwanted pregnancy is the main reason women seek abortion services, it is expected that induced abortionwill be common among women living with HIV, known to have a high prevalence of unintended pregnancy [15, 16].

However, studies examining the prevalence of induced abortion among women living with HIV are scarce. The few existing studies are mainly from the global north, indicating a clear gap in sub-Saharan Africa literature on abortion among women living with HIV. Existing studies, however, appear to suggest that the prevalence of induced abortion is higher among women living with HIV compared to HIV-negative women [10, 23]. For instance, Pinho et al. [24] found that the prevalence of induced abortion was 11.9% among women living with HIV/AIDS and 3.0% among women not living with HIV/AIDS. Another study also found that the prevalence of induced abortion among women living with HIV/AIDS was 6.5%, while women not living with HIV/AIDS was 2.9% [23]. Older age, higher education level, multiple sexual partnerships, having had children prior to the index pregnancy, and living with a sexual partner during pregnancy were identified as factors associated with the high prevalence of induced abortion among women living with HIV [23, 24].

Although the choice on termination on pregnancy Act 1996 gives South African women, regardless of age or marital status, the right to access comprehensive abortion services within the first 12 weeks of pregnancy, there are still challenges women face in procuring abortion, such as social stigma, thus, making women patronize unsafe backstreet induced abortions [25, 26]. Notwithstanding the restrictive socio-cultural environment in South Africa, women have the safe option of terminating a pregnancy. Research among pregnant women living with HIV who sought abortions has indicated problems they encounter when accessing safe abortion services, including faulty information and discriminatory treatment from healthcare providers [20, 21, 27]. Studies in South Africa have cited delays in healthcare provision and stigma as significant factors that hinder women living with HIV from seeking safe abortion care services [21, 22]. A few women were misinformed that only one legal abortion is allowed per woman [21]. A report by the South African National AIDS Council cited sterilization as a requirement from HIV-positive parturient women for obtaining a legal pregnancy termination [28].

To the best of our knowledge, there is not study that has estimated the prevalence and correlates of induced abortion among women living with HIV in South Africa. Our study fills this gap by examining the prevalence and correlates of induced abortion among parturient women living with HIV in South Africa. The findings of our study could inform health policies and planning regarding the provision of abortion care in South Africa (Tables 1 and 2).

**Table 1:** Demographic characteristics and prevalence of induced abortion among women living with HIV, 2016

Variables	All participants	Never induced abortion	Ever induced abortion
All	1709 (100.0)	1384 (81.0)	325 (19.0)
Age			
15–24 years	391 (22.9)	348 (89.0)	43 (11.0)
25 years and over	1318 (77.1)	1036 (78.6)	282 (21.4)
Education level			
Grade 8 or less	120 (7.0)	97 (80.8)	23 (19.2)
Grade 9 to12	1479 (86.5)	1198 (81.0)	281 (19.0)
Tertiary education	110 (6.4)	89 (80.9)	21 (19.1)
Marital status			
Ever married or cohabiting	522 (30.5)	383 (73.2)	140 (26.8)
Never married	1187 (69.5)	1002 (84.4)	185 (15.6)
Emploment status			
Employed	432 (25.3)	349 (80.8)	83 (19.2)
Unemployed	1277 (74.7)	1035 (81.0)	242 (19.0)
Place of residence			
Rural	585 (34.2)	480 (82.1)	105 (17.9)
Peri-urban	792 (46.3)	639 (80.7)	153 (19.3)
Urban	332 (19.4)	265 (79.8)	67 (20.2)
Parity			
1–2 children	1136 (66.5)	941 (82.8)	195 (17.2)
3–8 children	573 (33.5)	443 (77.3)	130 (22.7)
Smoking status			
Smoked during pregnancy	100 (5.9)	81 (81.0)	19 (19.0)
Quit smoking during pregnancy	80 (4.7)	56 (70.0)	24 (30.0)
Never smoked	1529 (89.5)	1247 (81.6)	282 (18.4)
Alcohol use			
Drank during pregnancy	235 (13.8)	193 (82.1)	42 (17.9)
Stopped drinking during pregnancy	431 (25.2)	343 (79.6)	88 (20.4)
Never drank alcohol	1043 (61.0)	848 (81.3)	195 (18.7)
HIV status at booking			
Positive	1383 (80.9)	1104 (79.8)	279 (20.2)
Negative or unknown	326 (19.1)	280 (85.9)	46 (14.1)

**Table 2:** Unadjusted and adjusted models showing the factors associated with induced abortion among women living with HIV, 2016

Variables	Model I uOR[95%CI]	Model II aOR[95%CI]
Age		
15–24 years	0.45 (0.32–0.64)***	0.51 (0.35–0.73)***
25 years and over	1	1
Education level		
Grade 8 or less	1.01 (0.52–1.94)	0.98 (0.51–1.93)
Grade 9 to12	0.99 (0.61–1.63)	0.95 (0.57–1.58)
Tertiary education	1	1
Marital status		
Ever married or cohabiting	1.99 (1.55–2.55)***	1.86 (1.43–2.41)***
Never married	1	1
Employment status		
Employed	1.02 (0.77–1.34)	0.92 (0.69–1.22)
Unemployed	1	1
Place of residence		
Rural	0.87 (0.62–1.22)	0.95 (0.66–1.36)
Peri-urban	0.95 (0.69–1.31)	0.96 (0.69–1.34)
Urban	1	1
Parity		
1–2 children	0.71 (0.55–0.91)*	0.96 (0.73–1.25)
3 to 8 children	1	1
Smoking status		
Ever smoked	1.39 (0.96–2.00)	1.51 (1.01–2.28)*
Never smoked	1	1
Alcohol use		
Ever drank	1.06 (0.82–1.35)	1.02 (0.78–1.35)
Never drank alcohol	1	1
HIV status at booking		
Positive	1.54 (1.10–2.16)*	1.44 (1.02–2.05)*
Negative or unknown	1	1

Exponentiated coefficients; 95% confidence intervals in brackets; *p < 0.05, **p < 0.01, ***p < 0.001; 1 = reference

## Methods

### Study design and setting

We analysed the baseline data of the East London Prospective Cohort Study, a cross-sectional study, which was conducted between September 2015 and May 2016 in three major maternity facilities in the Buffalo/Amathole districts of the Eastern Cape Province of South Africa [9, 29]. These health facilities serve a total population of 1,674,637 residents, with the Amathole district (892,637) marginally outnumbering the Buffalo City metropolitan area (755,000) [32]. The prevalence of HIV was 12.7% in the general population and 30% among pregnant women in the study setting [32]. The three health facilities chosen reflect the province’s diverse demographics and levels of health care. For example, Frere hospital is an urban tertiary health facility that receives patients from all over the province, while Cecilia Makiwane hospital, a regional health facility, is situated in the semi-urban Mdantsane township and offers both levels one and two services in the region. Bisho Hospital is a district health facility that mainly serves rural communities in Bisho and the surrounding areas. The study’s detailed methodology has already been published in previous studies [9, 16].

### Participants and sample size

The East London Prospective Cohort study was established to track PMTCT outcomes and associated behavioural factors with a view to improving quality of care in the study setting. It is made up of women diagnosed with HIV receiving maternity care in three health facilities. The sample size for the East London Prospective Cohort study was estimated using the approximate proportion of parturient women living with HIV on treatment one year after delivery in the study population [30]. Parturient women means mothers who are in labour or just gave birth. After adjusting for a likely 10% failure of follow-up within the first six months after delivery and allowing for a confidence interval of 2.5 percent with a confidence level of 95 percent, a sample size of 1709 participants was estimated for the study. In essence, the sample size was representative of the Buffalo/Amathole districts in the Eastern Cape Province, South Africa, since the study participants were recruited from three large clinics that cover these two districts. During the study period, all women living with HIV who delivered their babies at the participating hospitals’ maternity centers were eligible to take part. There was no refusal from the mothers. The data was collected using a questionnaire that had been pre-validated. It was tested on a group of 20 women who were not part of the main research. The questionnaires were administered by trained research assistants using the face-to-face interview technique. The research assistants had received training on ethical principles in research and interview techniques. Data obtained were captured into an electronic database to facilitate a follow-up study.

### Variables

#### Outcome variable

The outcome variable for this study was a self-reported lifetime induced abortion. Women were asked if they have ever induced abortion and their responses were Yes/No. All those who responded Yes were coded as “1’ and those who responded No were coded as “0”.

#### Independent variables

Nine independent variables were considered in the study. The variables were included based on previous studies on factors associated with induced abortion [22, 31].These comprised ages (15–24, 25 and above), educational level (grade 8 or less, grade 9–12, tertiary education), marital status (Never married, Ever married or cohabiting), employment status (employed, unemployed), place of residence (rural, peri-urban, urban), parity (1–2 children, 3–8 children), smoking status (smoking during pregnancy, quit smoking during pregnancy, never smoked), alcohol use (drank during pregnancy, stopped drinking pregnancy, never drank alcohol) and HIV status at booking (Positive, Negative or unknown).

### Data analyses

Frequencies and percentages were used to describe the participants’ sociodemographic characteristics and induced abortion. We fitted two binary logistic regression models. Model I was an unadjusted model that assessed the association between each of the independent variables against the outcome variable (induced abortion). The second model (Model II) was the adjusted model that contained all the independent variables and their association with induced abortion. The Model I and Model II results were presented as unadjusted odds ratios (uOR) and adjusted odds ratios (aOR), respectively, with their corresponding 95% confidence intervals indicating the estimates’ level of precision. The study employed binary logistic regression analysis because the outcome variable was dichotomous. In all the analyses, statistical significance was set at a p-value less than 0.05. Variance inflation factor was used to assess multicollinearity among the variables. All the analyses were carried out by using the IBM SPSS Statistics for Windows, Version 27.0 (IBM Corp., Armonk, New York, USA).

### Ethical consideration

We followed various steps to ensure the study adhered to all the ethical principles in line with the Declaration of Helsinki. First, the Walter Sisulu University Ethics Committee (reference number 098/2014) and the Eastern Cape Department of Health gave their approval upon assessing the research protocol before the data collection commenced. Second, the study procedures were approved by the management of all the three hospitals. Furthermore, the study’s purpose and process were explained to all the participants in an information sheet written in English and translated into IsiXhosa. Each participant gave written, informed consent to indicate her voluntary participation in the study. We obtained parental consent for three participants below 16 years, who assented to participate in the study. We respected the rights of participants to privacy, confidentiality, and anonymity throughout the study.

## Results

### Descriptive findings

The average age of participants was 29.63 ± 6.17 years. Most participants were over 25 years (77.1%), had grade 9 to 12 level of education (86.5%), unemployed (74.7%), and never married (69.5%). The prevalence of induced abortion was 19% (95% CI: 17.2–20.9%) but varied by sociodemographic characteristics. Induced abortion prevalence was higher among women aged 25 years and over (21.4%), ever married (26.8%), those already diagnosed positive before their index pregnancy than among women aged less than 25 years, never-married women, and those diagnosed with HIV during their index pregnancy.

### Multivariable findings

In the unadjusted logistic regression model, age 15–24 years, ever married, parity 1–2, and being diagnosed with HIV before the index pregnancy were significantly associated with having ever (lifetime) induced abortion. Education, employment, place of residence, and alcohol use were not significantly related to ever having an induced abortion. In the adjusted logistic regression model, all variables that were significant in the unadjusted regression maintained their direction and magnitude of effects, except for parity. Adolescents and young adults (aOR:0.51; 95% CI:0.35–0.73) had lower odds of having ever induced abortion, compared with women aged 25 years and above. Ever married or cohabiting women(aOR:1.86; 95% CI:1.43–2.41) had higher odds of having ever induced abortion compared with never-married women. Women who had one or two children were less likely to have induced abortion in comparison to those with three or more children. Women who ever smoked (aOR: 1.51; 95% CI: 1.01–2.28) were more likely to have induced abortion compared with those who never smoked. Women diagonosed positive with HIV before their index pregnancy (aOR:1.44; 95% CI: 1.02–2.05) were more likely to have induced abortion relative to those diagnosed during their index pregnancy.

## Discussion

The present study sought to assess the prevalence and factors associated with induced abortion among women living with HIV from three large health facilities in South Africa. This is the first study to examine the prevalence and factors associated with induced abortion among women living with HIV in South Africa to the best of our knowledge. We found that the prevalence of induced abortion was 19% but varied by sociodemographic characteristics. Our study’s prevalence of induced abortion suggests that the permissive legal context of abortion in South Africa may have helped some women access abortion upon demand [25, 32]. The findings from this study are vital in the sense that about 19 out of every 100 women who participated in this study have ever had an induced abortion. This reiterates the point that a greater proportion of these women need comprehensive abortion care services. It also draws attention to the fact that most of these women are experiencing unintended pregnancies, and hence their need for abortion care. Therefore, the more we report on the number of women living with HIV having abortions, the more healthcare providers will begin to understand that these women need abortion-related care. As such, results on abortion among this core group can help plan the various steps necessary for abortion care for the teeming population of women who need the service in the country.

The prevalence of induced abortion reported in the current study is lower than what was reported in previous studies in Brazil (30%) [33] and Italy (41.4%) [34]. It is worth noting that data of these studies were collected before the existence of highly active antiretroviral therapy (HAART) and when the vertical transmission prevention protocols were still starting, which restricts the comparability of the results to our study. Prevalence of induced abortion would expectedly drop significantly after the availability of HAART. Darak, et al. [31] studied 158 pregnancies of women after their HIV diagnoses and found that 51% were unwanted and 50% were induced abortion. The prevalence of induced abortion in our study is higher than what Pilecco et al. [22] and Pinho et al. [23], found (6.5% and 11.9%, respectively) among women living with HIV in Brazil. But these studies evaluated all pregnancies in contrast to our study that evaluated lifetime prevalence of abortion.

Another significant finding of this study is that adolescents and young adults were 49% less likely to have ever induced abortion compared with women aged 25 and above. This finding is consistent with the literature [35, 36]. This result is expected given that we measured the lifetime prevalence of induced abortion. Older people have more years of exposure to unintended pregnancies compared to younger ones due to their duration of sexual experience.

We also observed no socioeconomic variations (education, residence and employment status) in terms of the odds of pregnancy termination. This result contrasts with several previous studies in other parts of Africa that have reported socioeconomic and geographical inequalities in access to reproductive health services [35, 37]. The possible explanation for our finding could be the free healthcare policy in South Africa and availability of abortion services at the district hospitals across the region that has taken off the financial burden for the poor and therefore eliminated disparities between and within various groups in terms of healthcare access [38, 39].

Our study also shows that married/cohabiting women had higher odds of having ever induced abortion compared with never-married women. This finding contrasts with a previous study among women without HIV that reported a higher prevalence among single women [36]. It is worth indicating that abortion is not the risk itself; however, it is the unintended pregnancy. Evidence also shows a high prevalence of unintended pregnancy among women living with HIV in South Africa [14–16]. Married and cohabiting women are perhaps more sexually active than single women. Their exposure to sex means that their risk of unintended pregnancy is higher than single women, especially if they are not using effective contraceptives consistently [31]. Another possible reason is that married and cohabiting women may be older than the unmarried ones and might already achieve their fertility desire and therefore not want another child. If a woman become pregnant when she had achieved her fertility desire, she would most likely terminate the pregnancy, unlike if she has not reached her fertility goal.

Although not statistically significant in the adjusted model, women with parity of one or two had lower odds of having ever induced abortion than those with three to eight children. A previous study among women with HIV reported a similar result [31]. This finding is not surprising because the fertility rate per couple is 2.4 children [40] in South Africa. Those with fewer children might want to have more compared to those who had fulfilled their fertility desire. Women who have reached their ideal family size may choose to terminate an unwanted pregnancy to restrict their family size [41].

Lastly, our study shows that women diagnosed with HIV before their index pregnancy had higher likelihood of having ever induced abortion compared to those diagnosed during their index pregnancy. The literature has reported mixed results on whether the prevalence of induced abortion is higher among women with or without HIV. While a study that analysed data from Nigeria and Zambia shows that the odds of induced abortion did not differ among women with and without HIV [42], evidence from a recent systematic review [10] and surveys [20, 23] reported higher prevalence among women living with HIV than those without HIV. Fear of transmitting the virus to the baby [20, 21], exposing the child to later stigma, and discrimination, and leaving a child orphaned [20, 21] may have contributed to the relatively higher prevalence of induced abortion among women living with HIV.

## Strength and limitations

The major limitation of the present study is that abortion is self-reported. Due to abortion related stigma, some women may have induced abortion but fail to report it. Therefore, the prevalence of induced abortion in our study is conservative. Also, our study design was cross-sectional, and due to this, it is highly impossible to establish causal relationships. Because our study relied on past abortion experiences relative to current socioeconomic, our findings on the association between socioeconomic characteristics and abortion should be interpreted with caution. It is possible that sub-group of women needing more interventions are not identified due to the limitation of our dataset. Despite these limitations, the strengths are also worth acknowledging. First, the sample size is relatively large, and this has given us the statistical power to estimate factors associated with induced abortion among women with HIV. Again, the study was conducted in three large hospitals with diverse respondents from different backgrounds.

## Conclusion

The prevalence of induced abortion among parturient women living with HIV who participated in this study is relatively high. This is an indication that the need for abortion is somewhat high in this group of women. The finding, therefore, highlights the need for concerted efforts from all stakeholders to address the unmet need for contraception among women living with HIV to prevent unintended pregnancy. It is pertinent to address all barriers hindering access to comprehensive abortion care to ensure women use approved health facilities for this important reproductive health service.

## Data Availability

Data will be made available by the corresponding author upon reasonable request.

## Abbreviations

cOR: Crude odds ratios
aOR: Adjusted odds ratio
CI: Confidence interval
